# Structure and Conformational Dynamics of DMPC/Dicationic Surfactant and DMPC/Dicationic Surfactant/DNA Systems

**DOI:** 10.3390/ijms14047642

**Published:** 2013-04-09

**Authors:** Zuzanna Pietralik, Rafał Krzysztoń, Wojciech Kida, Weronika Andrzejewska, Maciej Kozak

**Affiliations:** Department of Macromolecular Physics, Adam Mickiewicz University, Umultowska 85, Poznań 61-614, Poland; E-Mails: zuzulka.p@wp.pl (Z.P.); ravkrz@gmail.com (R.K.); wk57@wp.pl (W.K.); staggioni@gmail.com (W.A.)

**Keywords:** phospholipids, dicationic surfactant, gemini surfactant, DNA, DNA-lipid interactions, DNA-surfactant interactions

## Abstract

Amphiphilic dicationic surfactants, known as gemini surfactants, are currently studied for gene delivery purposes. The gemini surfactant molecule is composed of two hydrophilic “head” groups attached to hydrophobic chains and connected via molecular linker between them. The influence of different concentrations of 1,5-bis (1-imidazolilo-3- decyloxymethyl) pentane chloride (gemini surfactant) on the thermotropic phase behaviour of 1,2-dimyristoyl-*sn*-glycero-3-phosphocholine (DMPC) bilayers with and without the presence of DNA was investigated using Fourier transformed infrared (FTIR) and circular dichroism (CD) spectroscopies, small angle scattering of synchrotron radiation and differential scanning calorimetry. With increasing concentration of surfactant in DMPC/DNA systems, a disappearance of pretransition and a decrease in the main phase transition enthalpy and temperature were observed. The increasing intensity of diffraction peaks as a function of surfactant concentration also clearly shows the ability of the surfactant to promote the organisation of lipid bilayers in the multilayer lamellar phase.

## 1. Introduction

Self-organization processes are crucial for biological activity of biomolecules such as lipids, nucleic acids and proteins [1,2]. These complicated phenomena are related to the formation of three-dimensional structures of proteins or nucleic acids, biological membranes and even more complicated structures of the macromolecular complexes like chromatin [3] or viral capsids [4]. Although self-organization process *in vivo* is complicated and still not fully understood [2], a better description of this process can be proposed on the basis of the model *in vitro* systems such as model lipid bilayers [5].

Membrane lipids as well as their derivatives and other amphiphilic molecules can form in water micelles and unilamellar or multilamellar vesicles, and in higher concentrations form lyotropic mesophases [5–7]. The self-association process of amphiphilic molecules is driven by the collective interactions between water, hydrophilic and hydrophobic parts of lipid molecules. The most significant interactions are between the hydrophobic groups, but electrostatic interactions between polar head groups and solvent, as well as hydrogen bond formation and geometry of the molecule, also play significant roles in the determination of symmetry and the stability of structures formed by lipids or surfactants [7,8].

Similar interactions induce DNA conformation to fold into well-defined secondary structures, such as A, B, Z or Ψ forms [9]. The stability of DNA structures also strongly depends on the presence of multivalent cations such as Mg^2+^ or spermine^4+^. These multivalent cations or polyamines are required to neutralize the negative charges of DNA and to decrease DNA-DNA repulsion forces [10]. DNA molecules in high concentrations form highly ordered mesophases *in vitro*, similarly to the other amphiplilic molecules [11].

An understanding of the interactions between DNA and polycationic molecules (e.g., cationic surfactants) as well as self-organization processes, can be used for the development of non-viral delivery systems for gene therapy [6]. Cationic lipids or surfactants are known for their ability to interact with DNA to form lipoplexes, lipid-DNA complexes with well-defined symmetry [10,12]. Lipoplexes are often formed on the basis of multilayer liposomes (MLV), with DNA molecules intercalated between lipid bilayers [12–14]. The nature of the strong interaction between cationic lipid bilayers and DNA molecules is based on electrostatic attraction, charge neutralization and an increase in entropy due to a release of counterions from DNA molecules intercalated between lipid bilayers [15]. This kind of tight binding implies successful protection of DNA molecules against the surrounding environment and has been used in the design of non-viral vectors for gene therapy. The promising group of amphiphiles, dicationic surfactants (also known as gemini surfactants) is currently under investigation for gene delivery purposes [16]. The molecule of a gemini surfactant is composed of two hydrophilic groups (polar “head-groups”) attached to hydrophobic chains and these moieties are interconnected by a molecular linker (at the level of “head-groups”). Their ability to form stable complexes with DNA in low concentrations and of relatively low toxicity make them the valuable components of non-viral vectors [16,17]. Moreover, their high conformational plasticity due to the polymethylene linker is often connected with formation of pH-induced H2c phase with DNA molecules [16–20]. This type of assembly is thought to have a significant role in cellular internalization during endocytosis [21].

Recently, it has been demonstrated that gemini surfactants containing an imidazolium moiety exhibit low toxicity and are promising systems in biomedical application as synthetic vectors for gene delivery [22]. This group of dicationic surfactants have a good binding capability to DNA molecules as demonstrated by agarose gel electrophoresis experiments [23]. Some of the dicationic surfactants based on imidazolium moiety have been reported to show the antimicrobial activity against Gram-negative bacteria *Escherichia coli*[24].

**Figure 1:**
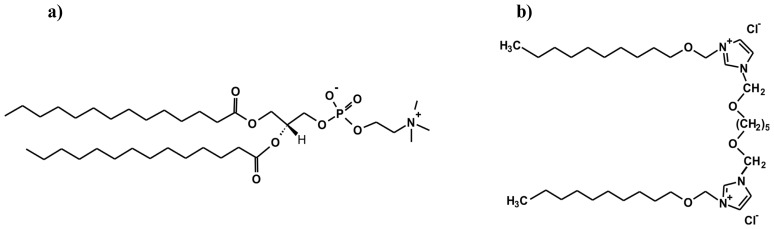
Chemical structures of 1,2-dimyristoyl-*sn*-glycero-3-phosphocholine (DMPC) (**a**) and 1,5-bis (1-imidazolilo-3-decyloxymethyl) pentane dichloride (**b**).

The aim of the studies performed was to check the influence of different concentrations of 1,5-bis (1-imidazolilo-3-decyloxymethyl) pentane dichloride (IMI_oxyC5_C10) (Figure 1) on the thermotropic phase behaviour of 1,2-dimyristoyl-*sn*-glycero-3-phosphocholine (DMPC) in mixed systems with and without DNA. The surfactant used in this study belongs to the group of dicationic surfactants (gemini surfactants) and has a geometry similar to that of a lipid molecule (two hydrophobic hydrocarbon chains). Such DMPC/gemini surfactant systems are prospective novel delivery systems for gene therapy [6].

## 2. Results and Discussion

### 2.1. Conformation of Lipid/Surfactant and Lipid/Surfactant/DNA Complexes

Fourier transformed infrared (FTIR) spectra obtained for DMPC/IMI_oxyC5_C10 and DMPC/IMI_oxyC5_C10/DNA complexes were dominated by vibrational bands of DMPC. The changes in vibrational modes reflect local effects at the molecular level, which in this certain type of structures are attributed to changes in structure and hydration level of lipid bilayers.

**Figure 2:**
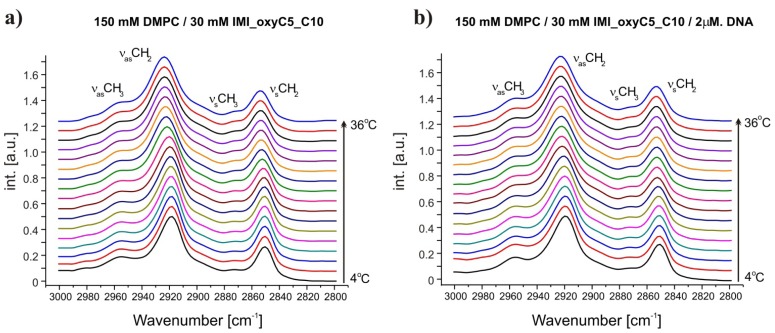
Selected Fourier transformed infrared (FTIR) absorption spectra in the spectral range of CH_2_ symmetric and asymmetric stretching vibrations at various temperatures. The spectra were obtained for DMPC/IMI_oxyC5_C10 (**a**) and DMPC/IMI_oxyC5_C10/DNA; (**b**) systems. Two additional bands of CH_3_ symmetric and asymmetric stretching vibrations are marked. The spectra are shifted for clarity.

**Figure 3:**
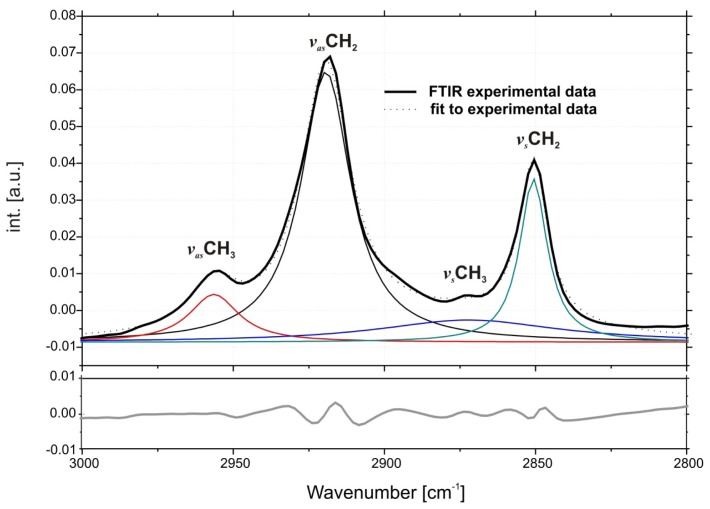
An example of deconvoluted spectrum in the region of CH_3_/CH_2_ symmetric and asymmetric stretching modes (top), bottom panel-residuals.

**Figure 4:**
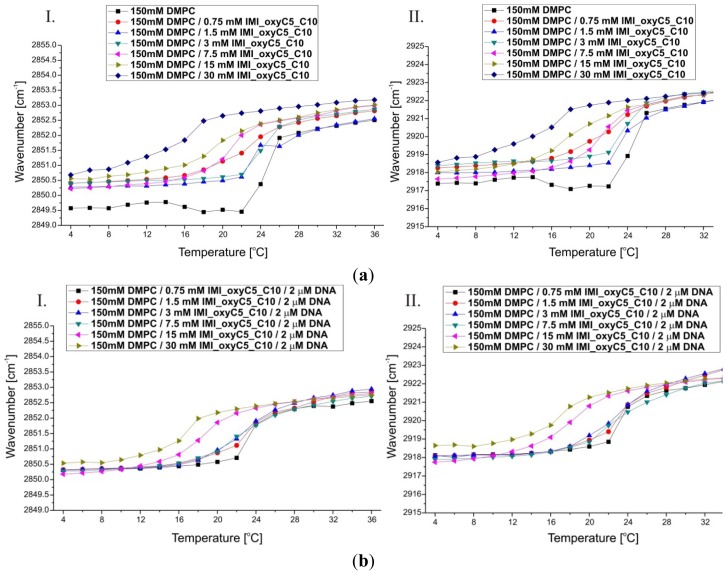
Thermal dependence of CH_2_ symmetric (I) and asymmetric (II) stretching modes. The wavenumber values are presented for selected DMPC/IMI_oxyC5_C10 (**a**) and DMPC/IMI_oxyC5_C10/DNA (**b**) samples.

Fully-hydrated DMPC forms three main types of lamellar mesophases present in the studied temperature range [25]. The phase transition sequence typical of DMPC Lβ′→Pβ′→Lα (gel→rippled gel→liquid crystalline) revealed upon increasing temperature, is manifested as discontinuous changes in band positions of CH_2_ symmetric (~2850 cm^−1^) and asymmetric (~2920 cm^−1^) stretching vibrations [26] and C=O stretching (~1734 cm^−1^) vibrations [27]. The exemplary FTIR absorption spectra from the CH_2_/CH_3_ stretching region collected for DMPC/IMI_oxyC5_C10 and DMPC/IMI_oxyC5_C10/DNA systems are presented in Figure 2. The example of deconvoluted spectrum in the spectral range of CH_3_/CH_2_ symmetric and asymmetric stretching vibrations is presented in Figure 3. The positions of CH_2_ stretching modes increase upon rippled gel to liquid crystalline phase transition (the main transition is often referred to as melting of the hydrocarbon chains of phospholipid) (see Figure 4). The spectral changes observed are directly connected with collective *trans-gauche* conformational transitions in hydrophobic polymethylene chains of DMPC molecules. The discontinuous increase in wavenumbers (~2 cm^−1^ and ~4 cm^−1^) in the positions of bands characteristic of symmetric and asymmetric CH_2_ stretching vibrations respectively, observed upon the heating of the samples, was in accordance with earlier observations [26].

The increase in IMI_oxyC5_C10 surfactant concentration led to a significant decrease in the main transition (Pβ′→Lα) temperature (~24 °C for 150 mM DMPC and ~18 °C for 150 mM DMPC/30 mM IMI_oxyC5_C10) as well as to a broadening of the temperature range of transition as shown in Figure 4. Moreover in the full temperature range a slight shift (by about 0.5–1 cm^−1^) of the wavenumbers characterising both CH_2_ stretching modes was noted with the increasing gemini surfactant concentration. These significant changes in phase behaviour of DMPC bilayers occurring with increasing surfactant concentration might be a result of internalisation of surfactant molecules into the bilayer structure. This mechanism leads to a main transition temperature shift to lower values as well as to a broadening of the transition temperature range observed for CH_2_ symmetric and asymmetric stretching band positions.

Such phase transition behaviour was characteristic also of other lipid/surfactant mixed systems including those containing anionic or cationic surfactants (such as sodium dodecyl sulfate, dodecyltrimethylammonium bromide [28] or (alcoxymethyl)dodecydimethylammonium chlorides [29,30]), zwitterionic surfactants (e.g., sulfobetaines [31–33]) as well as dicationic surfactants (alkane-α,ω-diyl-bis(dodecyldimethylammonium bromides) [34], 1,1′-(1,6-hexan)bis-3- octyloxymethyl-imidazolium di-chloride [35]).

The temperature dependence of band positions of CH_2_ stretching vibrations for DMPC/IMI_oxyC5_C10/DNA complexes (Figure 4) was comparable to that described above for DMPC/surfactant systems containing the same surfactant concentrations. Previous studies of cationic DMPC/DOTAP systems with a low DNA proportion observed that DNA insignificantly perturbs the global lipid organization or induced only a small disordering effect (with higher DNA concentrations) on the DMPC acyl chains [36]. These observations suggest that only weak interactions take place between the lipid acyl chains and DNA, which is in very good agreement with our results.

**Figure 5:**
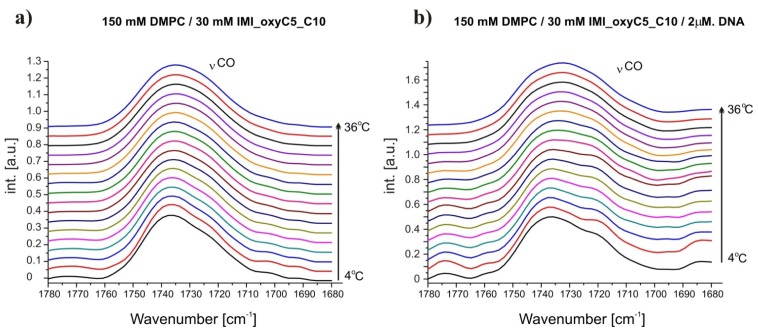
Selected FTIR absorption spectra in the spectral range of C=O stretching vibration at various temperatures obtained for DMPC/IMI_oxyC5_C10 (**a**) and DMPC/IMI_oxyC5_C10/DNA; (**b**) systems. The spectra are shifted for clarity.

Carbonyl groups of phospholipids present at the interface, between hydrophobic and hydrophilic parts of the bilayer, are capable of forming a hydrogen bond network with surrounding water molecules [37,38]. This fact leads to formation of two populations of carbonyl groups: water-bounded and unbounded. In the FTIR spectrum, these interactions are manifested by peak splitting into two bands, each connected with one of the described state of C=O group. Positions and intensities of those peaks vary for different lamellar mesophases (Lβ′ phase: ~1730.7 cm^−1^—water-bounded state, ~1738.4 cm^−1^—water-unbounded state; Pβ′ phase: ~1724 cm^−1^—water-bounded, ~1742 cm^−1^— water-unbounded; Lα phase: ~1721.5 cm^−1^—water-bounded, ~1738 cm^−1^—water-unbounded; as referred in [27]) Therefore, the changes observed for C=O stretching vibrations provided information about the hydration level of bilayers. The hydration level of bilayers increases with increasing temperature [26,27]. For effective analysis of hydration changes, C=O stretching bands were localised using the first spectral momentum of the band (the centre of gravity) (Figure 5). This method allows investigation of collective band changes treated as the effect of both, intensity and peak positions variations.

**Figure 6:**
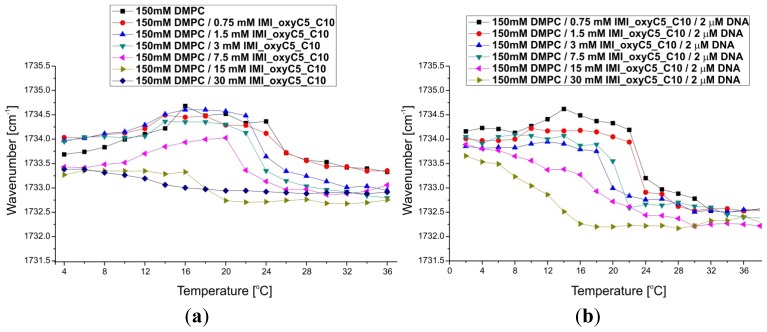
Thermal dependence of C=O stretching band positions for DMPC/IMI_oxyC5_C10 (**a**) and DMPC/IMI_oxyC5_C10/DNA; (**b**) systems.

Two changes in the carbonyl stretching band position were observed in the spectrum of the reference system—150 mM DMPC water solution (Figure 6). The observed shift in the *v*_CO_ band position towards higher wavenumbers (~0.6 cm^−1^) reflects the appearance of pretransition (Lβ′→Pβ′) in the sample and the subsequent shift towards lower values (~1.5 cm^−1^) is directly related to the main phase transition. The increase in the surfactant concentration in the systems studied led to a decrease in the transition temperature characterising the pretransition and the main transitions, and also to broadening of their temperature ranges. Spectral changes connected with the pretransition disappeared for two highest concentrations of gemini surfactant (15 mM and 30 mM IMI_oxyC5_C10). In the absorption spectra of these two systems only a single shift towards decreasing wavenumbers values ~0.7 cm^−1^ was noted. Additionally, at high concentrations of the surfactant (7.5 mM, 15 mM and 30 mM IMI_oxyC5_C10) the carbonyl stretching band was shifted towards decreasing wavenumbers in the full temperature range.

The mechanism of pretransition depends on two events [39]—the change in the tilt angle of hydrophobic chains and the changes in the effective area of hydrophilic head group as a result of increasing hydration, which in consequence leads to formation of a characteristic rippled structure. The presence of IMI_oxyC5_C10 surfactant in the bilayer structure may affect this mechanism in several ways. Electrostatic repulsion between the cationic head groups of the surfactant leads to an increase in the effective area of head groups and loosens up the packing of molecules in the bilayer. The increased orientation freedom may affect the collective hydrophobic chain tilt, and in consequence, the lateral stress of hydrophobic chains, responsible for formation of ripples, will be reduced. Moreover, the rippled gel phase stability depends on fluctuational correlations between adjacent bilayers [39]. For higher surfactant concentrations (the two samples of the highest IMI_oxyC5_C10 surfactant concentration) the correlations may disappear as a consequence of electrostatic repulsions between bilayers [39]. These structural disruptions are connected also with the increase in bilayer hydration. Facilitated water penetration into the bilayer interphase was observed as the C=O stretching band was shifted towards smaller wavenumbers upon increasing surfactant concentration.

Similarities between the FTIR spectra recorded for DMPC/IMI_oxyC5_C10/DNA and DMPC/IMI_oxyC5_C10 were also noted in the region of the stretching vibrations of the carbonyl group. In particular, the temperatures of phase transitions and the thermal ranges of these transitions were close. The only significant differences concerned the positions of the bands corresponding to the stretching vibrations of C=O in the liquid crystalline phase (Lα), for DMPC/IMI_oxyC5_C10/DNA as they appeared at by about 0.5 cm^−1^ lower wavenumbers than in the spectra of DMPC/IMI_oxyC5_C10.

Moreover for DMPC/IMI_oxyC5_C10/DNA lipoplexes, the characteristic shift of the C=O stretching band positions towards increasing wavenumbers (directly related to the pretransition) disappeared at 3 mM concentration of the surfactant in the system studied, and did not appear for any higher surfactant concentration (Figure 6).

**Figure 7:**
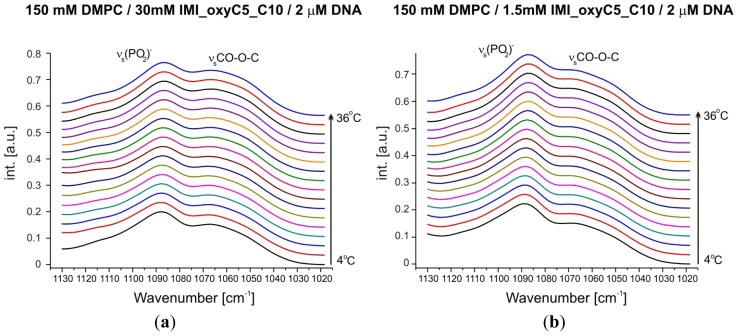
Selected FTIR absorption spectra in the spectral range of (PO_2_)^−^ symmetric stretching vibrations at various temperatures and different surfactant concentrations (**a**,**b**) obtained for DMPC/IMI_oxyC5_C10/DNA systems. An additional band of CO–O–C symmetric stretching vibration was observed. The spectra are shifted for clarity.

**Figure 8:**
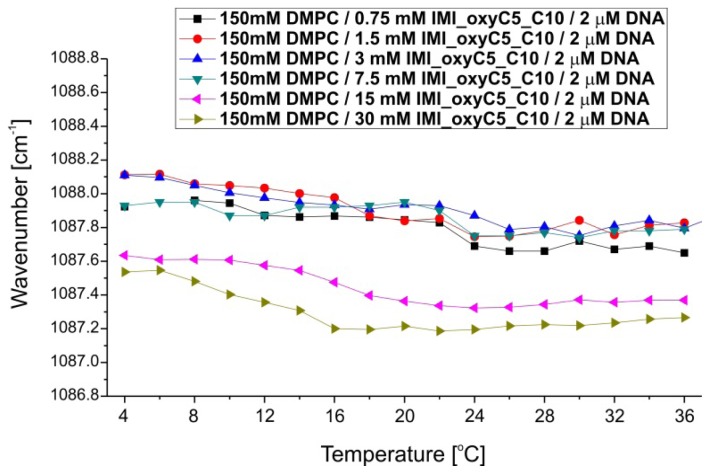
Thermal dependence of (PO_2_)^−^ symmetric stretching band positions for selected DMPC/IMI_oxyC5_C10/DNA systems.

The FTIR spectra demonstrated also changes in the (PO_2_)^−^ symmetric stretching region (Figures 7 and 8). Phosphate groups are components of DMPC (the dominating population) as well as DNA molecules. Samples with the highest two concentrations of IMI_oxyC5_C10 surfactant (15 mM and 30 mM) show in full temperature range ~0.4 cm^−1^ shift towards lower wavenumbers (Figure 8). Moreover, for these samples, the temperature dependence of the (PO_2_)^−^ stretching band position revealed the stepwise shift (~0.4 cm^−1^ at around *T* = 16 °C) upon temperature increase. These changes correspond to the shift of the C=O stretching band position towards decreasing wavenumbers observed for these samples.

**Figure 9:**
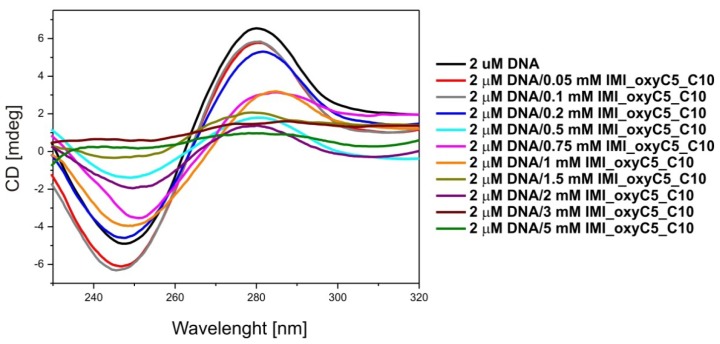
Circular dichroism spectra of DNA/IMI_oxyC5_C10 lipoplexes (0.05–5 mM IMI_oxyC5_C10).

Circular dichroism is the most popular spectroscopic method used for monitoring structural changes in the nucleic acid conformation. Therefore, circular dichroism was selected as the most sensitive method to determine the influence of IMI_oxyC5_C10 on DNA conformation. The CD spectra of pure DNA and lipoplexes with 0.05–5 mM of IMI_oxyC5_C10 are presented in Figure 9. The spectrum of the pure DNA solution exhibits a positive band near 277 nm, a negative band near 245 nm and a crossover point near 260 nm, indicating a right-handed B-DNA form [40,41], which is a typical native double-stranded conformation of fully hydrated DNA.

The increased surfactant concentration slightly shifted the bands towards higher wavelength, for the negative band to 252 nm and for the positive band to 284 nm. The addition of the surfactant studied resulted in the interaction between the positively charged groups of the surfactant with the polyanionic DNA molecule, causing the exposure of hydrophobic parts of the surfactant to the solution.

The changes in the intensity of the CD bands can be assigned to changes in the hydration shell of the phosphate groups of DNA [42]. The results obtained indicate that the DNA maintains the B-form up to the 2 mM concentration of IMI_oxyC5_C10 surfactant in the solution.

In complexes of DNA with dodecyltrimethylammonium bromide (DTAB), the almost identical shifts of the negative and positive bands in the CD spectrum were not accompanied by the conformational transition from B to A or B to Z form [43]. Similar interactions between DNA and some polyamines, (e.g., spermine), observed in the CD spectra were described by Chang *et al.*[44]. Therefore, these changes can be attributed to the local perturbations in the DNA base geometry rather than to a change in the DNA helical structure. Higher concentration of IMI_oxyC5_C10 surfactant induces precipitation of IMI_oxyC5_C10/DNA lipoplexes. Unfortunately, due to the high absorption of polarised light in the UV range by phospholipids, the examination of DMPC/surfactant/DNA systems using circular dichroism was not possible.

### 2.2. Thermal Stability of Lipid/Surfactant and Lipid/Surfactant/DNA Complexes

**Figure 10:**
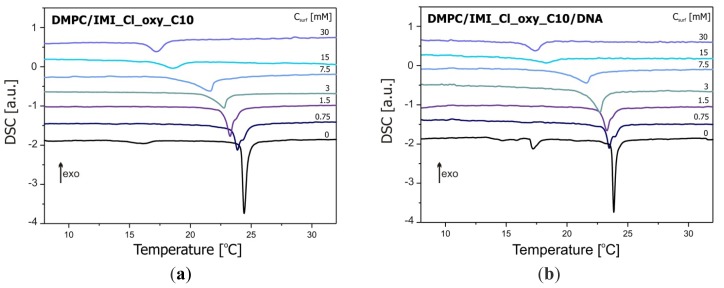
DSC thermograms of DMPC/IMI_oxyC5_C10 (**a**) and DMPC/IMI_oxy C5_C10/DNA; (**b**) systems.

The results of DSC studies of DMPC/dicationic surfactant and DMPC/dicationic surfactant/DNA systems are shown in Figure 10, while the parameters characterising the phase transitions obtained from these data are summarised in Table 1. The addition of IMI_oxyC5_C10 surfactant and DNA has a pronounced effect on the thermodynamic parameters of phase transitions observed for DMPC. This effect is however much stronger upon the surfactant addition. For the reference DMPC system, two phase transitions are observed and characterised by *T*_onset_ = 14.8 °C and *T*_onset_ = 24.2 °C corresponding to pretransition and main transition, respectively. These values are in agreement with the corresponding ones obtained for pure hydrated DMPC solutions [45].

The influence of the dicationic surfactant on the thermodynamic parameters was significant. Even the smallest amount of IMI_oxyC5_C10 surfactant in the systems studied (0.75 mM) resulted in complete disappearance of the pretransition and a significant shift of the main phase transition temperature (Δ*T* = −0.7 °C). Simultaneously, a splitting of the main transition peak was observed. The first maximum can be assigned to the main transition process of DMPC/dicationic surfactant mixed phase, while the weak second peak corresponded to the main transition of pure DMPC. This effect disappears at IMI_oxyC5_C10 surfactant concentrations above 1.5 mM. With increasing concentration of IMI_oxyC5_C10 surfactant, a decrease in the main phase transition enthalpy and temperature was observed.

A decrease in enthalpy characterising the main phase transition in the systems with the highest surfactant concentrations (15 and 30 mM of IMI_oxyC5_C10) in relation to pure DMPC reached even 65% (ΔΔ*H* = 10.8 kJ/mol) and 62% (ΔΔ*H* = 10.3 kJ/mol). The addition of DNA to the pure phospholipid solution caused a small reduction in temperature (Δ*T* = −0.5 °C) and enthalpy of the main phase transition (ΔΔ*H* = −0.6 kJ/mol).

The pretransition was detected only for the reference system DMPC (Δ*H* = −2.3 kJ/mol, *T*_onset_ = 14.8 °C and *T*_peak_ = 16.1 °C) and for the system DMPC/DNA (Δ*H* = −3.6 kJ/mol; *T*_onset_ = 17.0 °C and *T*_peak_ = 17.2 °C). After addition of the gemini surfactant, the pretransition is undetectable in the two series of samples studied (with and without DNA). These effects are also in full agreement with the earlier discussed FTIR results for these systems.

For the complexes of DMPC/IMI_oxyC5_C10/DNA, the decrease in enthalpy of the main phase transition was even a bit greater, while the shift of the characteristic temperatures was a bit smaller than for DMPC/IMI_oxyC5_C10 systems (see Table 1). The temperature changes related to the incorporation of DNA and surfactant molecules into the lamellar phase have been observed for other systems of this type [46].

### 2.3. Structural Parameters of Lipid/Surfactant/DNA Complexes

**Figure 11:**
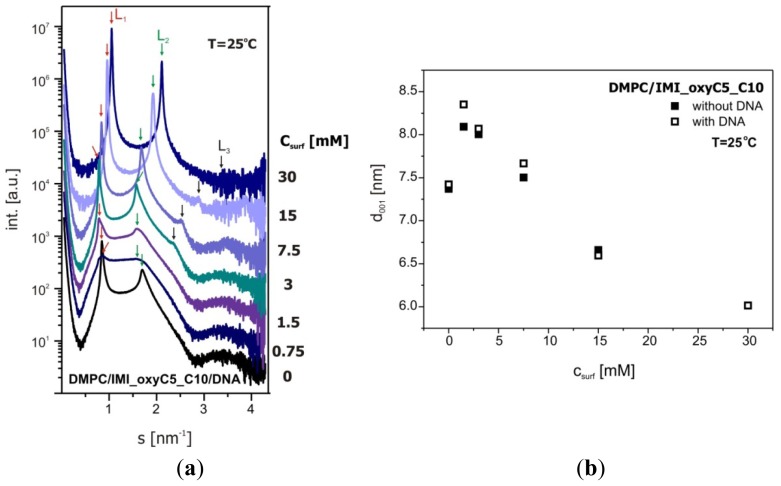
SAXS data collected for DMPC/IMI_oxyC5_C10/DNA systems (**a**) and d-spacing (d_001_) characterising these systems (**b**).

Figure 11 presents exemplary SAXS data recorded for DMPC/IMI_oxyC5_C10/DNA systems and d-spacing (d_001_), calculated on the basis of scattering patterns. It should be noted that the concentration of DNA in the systems tested was relatively low in comparison to that in typical lipid/DNA model systems. The DNA concentration in the sample was chosen so that the ratio of negative charge of DNA polyanion (assuming a DNA mean chain length as 170 bp), to the positive charge of IMI_oxyC5_C10 surfactant ranged from 1.0 to 0.02. These values correspond to the typical range of DNA concentrations tested for the cationic carriers used in gene therapy.

Considering the low DNA concentration, we did not expect dramatic structural changes in the lipid matrix induced by the presence of DNA. The observed changes (about 0.4 nm) of the bilayer spacing were small in comparison with those in the systems not containing DNA. The observed increasing intensity of the diffraction peaks as a function of surfactant concentration also clearly shows the ability of the surfactant to promote organisation of lipid bilayers in the multilayer lamellar phase. Moreover, with increasing concentration of the surfactant in the system DMPC/IMI_oxyC5_C10/DNA (from 3 mM IMI_oxyC5_C10) besides the diffraction maxima *L*_1_ and *L*_2_ corresponding to the lattice constants d_001_ and d_002_, the SAXS curves reveal the third diffraction maximum, *L*_3_ (see Figure 11a). Detailed analysis of the d-spacing corresponding to this peak and its relation to the lattice constants d_001_ and d_002_, has clearly confirmed the presence of lamellar phase in the whole range of IMI_oxyC5_C10 concentrations studied in the systems of interest in this work. It should be emphasised that the above described structural changes manifested in the SAXS curves correlate well with the changes in the C=O stretching band positions as a function of the surfactant concentration (see Figure 6). The correlations between SAXS and FTIR results support the conclusion that the presence of the surfactant promotes ordering of DMPC and DNA in the lamellar phase.

In previous studies conducted by Pullmannová *et al.*[34] or Munoz-Ubeda *et al.*[47], the diffraction peaks associated with the presence of DNA were also reported. In the system studied in this work, the DNA concentration was 10 times lower and the characteristic peak related to DNA-DNA packing [43] was not observed. In the DMPC/IMI_oxyC5_C10/DNA system the increasing concentration of surfactant in the system caused a clearly visible decrease in the d-spacing value. Similar effects that the repeat distance d_001_ decreases linearly with increasing molar ratio surfactant/phospholipid were observed for gemini surfactants-DOPE-DNA complexes [34].

**Figure 12:**
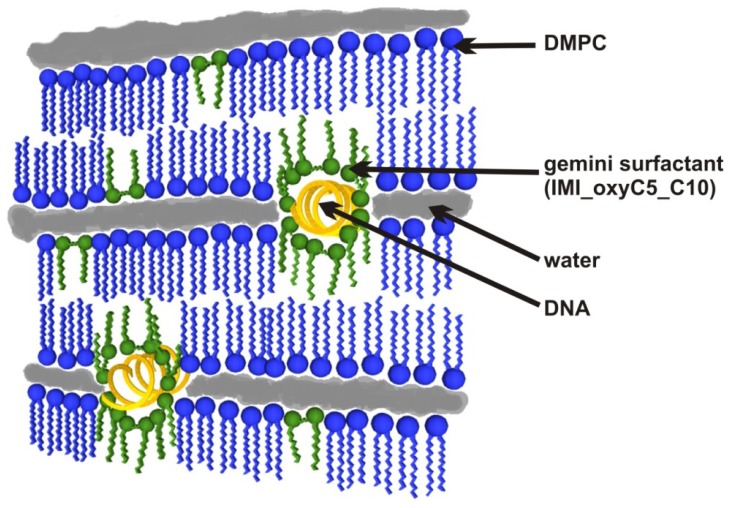
The structural model of DMPC/IMI_oxyC5_C10/DNA in the lamellar phase.

On the basis of the typical symmetry of diffraction patterns clearly showing the lamellar nature of the systems investigated, and on the basis of the previously described patterns of lipid-DNA interactions [48–51], a proposed scheme of DMPC/IMI_oxyC5_C10/DNA lipoplexes structure is shown in Figure 12.

## 3. Experimental Section

### 3.1. Sample Preparation

1,2-dimyristoyl-*sn*-glycero-3-phosphocholine was obtained from Avanti Polar Lipids, Inc. (Alabaster, AL, USA) as lyophilized powders. Dicationic (gemini) surfactant 1,5-bis(1-imidazolilo-3- decyloxymethyl) pentane dichloride (IMI_oxyC5_C10) was synthesized according to the synthesis method reported by Pietralik *et al.*[52]. Deoxyribonucleic acid of low molecular weight from salmon sperm was purchased from Sigma-Aldrich and used without further purification.

The homogeneous 150 mM solution of DMPC in 20 mM sodium citrate/D_2_O pH 6.5 and DMPC/IMI_oxyC5_C10 systems containing 150 mM DMPC and 0.75, 1.5, 3, 7.5, 15 and 30 mM of IMI_oxyC5_C10 were prepared by 10 cycles of sonication at 30 °C for 30 minutes and cooling at 4 °C. The DMPC/IMI_oxyC5_C10/DNA systems were prepared on the basis of lipid/surfactant systems described above, but with twice the concentrations of phospholipid and surfactant. Then lipid/surfactant solutions were mixed in a volume ratio of 1:1 with DNA solution (4 M), so that the final DNA concentration was 2 μM.

### 3.2. FTIR Measurements

The infrared spectra were obtained by the attenuated total reflection (ATR) technique using a Tensor 27 (Bruker Optics, Ettlingen, Germany) spectrometer with a horizontal Platinum ATR device (diamond, 45 deg.). Samples of 0.02 cm^3^ volume were placed on diamond window, enclosed by a heating cover and incubated for 15 min at a given temperature before starting the experiment. The data were collected at temperatures ranging from 4 to 36 °C. For each spectrum, 256 scans in the spectral range of 4000–600 cm^−1^ were taken. The resolution of the experimental data was 2 cm^−1^. The temperature was controlled by a Haake DC-30 temperature unit. The data were analysed using the Opus (Bruker Optics, Ettlingen, Germany) program package. The positions of characteristic bands were obtained using spectra second derivative.

### 3.3. Circular Dichroism (CD)

Electronic circular dichroism (CD) spectra were collected using J-815 spectropolarimeter (Jasco, Japan). CD spectra were collected at 2 μM DNA concentration and in the presence of IMI_oxyC5_C10 (concentrations from 0.05 to 5 mM). Scans were taken in the ultraviolet (UV) CD region (220–340 nm) with a bandwidth of 1 nm, at a scanning rate 50 nm/min and using a standard quartz cell of 0.5 mm path length.

Each spectrum was obtained by averaging of 10 runs at a fixed temperature of 25 °C. The CD spectra obtained were processed using the Spectra Manager II software (Jasco, Japan).

### 3.4. Differential Scanning Calorimetry (DSC)

The DSC measurements were performed using a DSC-204 Phoenix Netzsch calorimeter equipped with a high sensitivity μ-sensor. The calorimetric system was calibrated, in transition temperature and enthalpy changes, by using cyclohexane, mercury, indium, bismuth, tin and biphenyl. The samples of about 20 mg were sealed in hermetic aluminium crucibles. All measurements were performed in helium atmosphere in temperatures from 5 to 35 °C at a scanning rate of 1 °C/min. The DSC data were processed and analysed using the TA (Neztsch) program. For determination of enthalpy values of the representative phase transitions, a linear base line was used.

### 3.5. Small Angle X-Ray Scattering (SAXS)

SAXS measurements were performed on P12 Beamline of EMBL Hamburg Outstation on PETRA III storage ring at DESY [53,54]. Scattering patterns were collected using a Photon counting Pilatus 2M pixel detector (253 × 288 mm^2^) at the sample to detector distance of 3500 mm. The scattering vector range was 0.08 > *s* > 4.5 nm^−1^. The detector *s*-axis (where *s* = 4πsinθ/λ with 2θ the scattering angle, wavelength = 0.1 nm) was calibrated using the diffraction patterns of silver behenate [55]. The measurements were carried out on a series of DMPC/surfactant/DNA solutions in 50 mM sodium citrate pH 7.0 in D_2_O containing the surfactant at increasing concentrations (0–30 mM). The SAXS data were collected in 20 successive 0.1 s frames. All measurements were performed using a capillary cell (sample volume 10 μL) and automated filling at 25 C. The collected frames were integrated and averaged. The scattering data were corrected for detector response, normalised to the incident beam intensity and the scattering of the buffer was subtracted using the program package PRIMUS [56].

## 4. Conclusions

The presence of DNA molecules in DMPC/IMI_oxyC5_C10 system did not affect the conformational dynamics of CH_2_ groups (symmetric and asymmetric stretching bands) and the *trans-gauche* transitions connected with the main phase transition. However, in the presence of the surfactant the temperature of the main phase transition was significantly shifted towards lower temperatures, which is well illustrated not only by FTIR but also by DSC data.

The pretransition in DMPC disappeared in all DMPC/surfactant complexes (with and without DNA). This phenomenon can be related to the incorporation of surfactant molecules in the lipid bilayer and the interactions of the surfactant polar groups with the polar components of DMPC. Moreover, the disturbances to the lipid bilayer can also be responsible for increasing level of hydration observed in the liquid crystalline phase (low C=O stretching band position) and the interactions of DNA with the lipid polar groups [57]. These conclusions are confirmed by the correlations between the changes in positions (wavenumbers) of the bands corresponding to the stretching vibrations of C=O and (PO_2_)^−^, which reflect the increased affinity between DNA and lipid bilayers in the liquid crystal phase.

The increased surfactant concentration slightly shifted the characteristic CD spectrum towards higher wavelengths, which indicates that the DNA maintains the B-form up to the 2 mM concentration of IMI_oxyC5_C10 surfactant in the solution. We can conclude also that the addition of IMI_oxyC5_C10 resulted in the interaction between the positively charged groups of the surfactant with the polyanionic DNA molecule, causing the exposure of hydrophobic parts of the surfactant to the solution. The changes in the intensity of the CD bands are connected with those in the hydration shell of the DNA phosphate groups.

On the basis of the SAXS and DSC data obtained for the DMPC/IMI_oxyC5_C10/DNA lipoplexes studied, indicating its ordering, structural stability and thermal stability, it is reasonable to conclude that this system can meet the requirements of non-viral carriers for DNA transfer in gene therapy.

## Figures and Tables

**Table 1:** The enthalpy and characteristic temperatures of main transition for DMPC/IMI_oxyC5_C10 and DMPC/IMI_oxyC5_C10/DNA systems.

DMPC/IMI_oxyC5_C10				DMPC/IMI_oxyC5_C10/DNA			
	
*C*_surf_ (mM)	*H* (kJ/mol)	*T*_peak_ (°C)	*T*_onset_ (°C)	*C*_surf_ (mM)	*H* (kJ/mol)	*T*_peak_ (°C)	*T*_onset_ (°C)
0	−16.7	24.4	24.2	0	−16.1	23.9	23.7
0.75	−12.2	23.9	23.4	0.75	−10.4	23.5	23.1
1.5	−13.1	23.3	22.9	1.5	−11.5	23.3	22.8
3	−9.6	22.8	21.5	3	−11.2	22.7	22.0
7.5	−10.0	21.6	20.4	7.5	−7.0	21.6	20.2
15	−5.9	18.5	16.9	15	−4.8	18.3	17.1
30	−6.4	17.2	16.4	30	−6.1	17.4	16.6
